# Assessment of Serum and Saliva CRP, IL-17, and IL-19 Levels in Patients with Different Severity of Acne Vulgaris

**DOI:** 10.3390/ijms262412117

**Published:** 2025-12-17

**Authors:** Nazlı Karimi Ahmadi, Okan Arıhan, Aslı Şan Dağlı Gül, Sedef Nur Yeşil, Başak Yalıcı Armağan

**Affiliations:** 1 Physiology Department, Faculty of Medicine, Hacettepe University, Ankara 06230, Turkey; okan.arihan@hacettepe.edu.tr (O.A.); aslisandagligul@hacettepe.edu.tr (A.Ş.D.G.); 2 Dermatology Department, Faculty of Medicine, Hacettepe University, Ankara 06230, Turkey; sedef.yesil@hacettepe.edu.tr (S.N.Y.); basakarmagan@gmail.com (B.Y.A.)

**Keywords:** acne vulgaris, interleukin-17, interleukin-19, C-reactive protein, salivary CRP

## Abstract

Acne vulgaris (AV) is a chronic inflammatory skin disorder, and cytokines such as interleukin-17 (IL-17), interleukin-19 (IL-19), and C-reactive protein (CRP) are thought to contribute to its immunopathogenesis. This study investigated serum and salivary levels of CRP, IL-17, and IL-19 in patients with AV and examined their relationship with disease severity. A total of 99 participants aged 15–30 years were classified into Control (n = 28), Moderate AV (n = 43), and Severe AV (n = 28) groups using the Global Evaluation Acne (GEA) Scale. Serum and saliva samples were analyzed using ELISA and immunoturbidimetric assays. Statistical comparisons and correlation analyses were performed. Serum IL-17 levels were significantly higher in the control group compared to both acne groups (*p* < 0.05), with no gender-related differences. Salivary cytokine levels showed no significant group differences. However, IL-17 and IL-19 were strongly correlated in both saliva (r = 0.672, *p* < 0.005) and serum (r = 0.538, *p* < 0.005) across the entire study population. Serum and salivary CRP levels showed no significant differences between groups. In contrast to previous reports, our study found lower serum IL-17 levels in AV patients compared to healthy controls, challenging the assumption of its purely pro-inflammatory role and suggesting a potential compensatory or regulatory immune mechanism to maintain homeostasis. These findings may also reflect distinct physiological pathways between systemic interleukin activity and localized skin inflammation. Although salivary cytokine levels did not differ significantly among groups, strong intra-sample correlations highlight their interaction and support saliva’s potential as a non-invasive tool for monitoring immune activity.

## 1. Introduction

### 1.1. Epidemiology

Acne vulgaris (AV) is an inflammatory disease of the pilosebaceous unit that primarily affects the face and trunk [1]. It is a common chronic inflammatory skin disease that affects approximately 9% of the global population and 80% of young adults [2]. Although comprehensive global updates remain limited, the most widely cited epidemiological review by Tan and Bhate (2015) reports that acne vulgaris affects up to 85% of adolescents and constitutes one of the most common dermatological conditions worldwide [3].

### 1.2. Clinical Features and Burden

AV is characterized by the presence of open and closed comedones, as well as inflammatory lesions such as papules, pustules, and nodules, primarily in areas with an increased density of pilosebaceous follicles [2]. Acne is typically a self-limiting condition, but its natural resolution can take several years. Moreover, moderate to severe cases have the potential to cause permanent scarring [4]. Acne negatively impacts quality of life and self-esteem and severe scarring acne is associated with an increased risk of anxiety, depression, suicidal thoughts [5,6].

### 1.3. Current Treatment Overview

Acne management is tailored according to disease severity and generally involves a combination of topical and systemic therapeutic options [7]. Topical agents remain the foundation of both induction and maintenance therapy, while systemic antibiotics, antiandrogen treatments, and oral isotretinoin are reserved for moderate to severe cases. In mild disease, topical retinoids, benzoyl peroxide, azelaic acid, or their combinations are considered first-line treatments. For moderate to severe AV, fixed-dose topical therapy is typically combined with systemic antibiotics, with isotretinoin or antiandrogens introduced when appropriate. Oral isotretinoin is regarded as first-line for very severe forms, including nodular, cystic, and conglobate acne [8]. After clinical improvement is achieved, maintenance therapy with a topical retinoid, with or without benzoyl peroxide, is recommended to reduce the risk of relapse.

Therapeutic approaches for acne vulgaris also vary according to overall disease complexity. As AV is a highly multifactorial inflammatory condition, adjunctive or supportive treatments that target inflammation and immune modulation play an important role alongside conventional therapies. These strategies can enhance treatment outcomes and contribute to more personalized management [9].

### 1.4. Microbiology and Pathophysiology

Clinical medicine recognizes acne as a complex, multifactorial disease, primarily driven by inflammation, immune response, sebaceous gland hyperplasia, and *Cutibacterium acnes* colonization. These interconnected factors play a crucial role in the development and progression of AV, highlighting the immune-inflammatory response as a key contributor [10,11,12,13]. *Cutibacterium acnes* is thought to trigger the inflammatory response in acne vulgaris (AV) through Toll-like receptors (TLRs) and may lead to exaggerated immune responses [14].

### 1.5. Inflammatory and Cytokine Pathways

Both innate and adaptive immunity, particularly Th17 pathways, play a key role in inflammatory response [10]. *Cutibacterium acnes* has been shown to stimulate IL-17A and IFN-γ production, enhancing inflammatory cytokine expression [15,16]. IL-17, secreted by activated immune cells, is believed to be crucial in various skin diseases [17,18]. Despite numerous studies, the etiopathogenesis of acne vulgaris (AV), especially in its severe and scarring forms, remains incompletely understood [19]. A 2019 study linked elevated serum IL-17 levels in AV patients to disease pathogenesis, suggesting its potential as a biomarker and predictor of severity and scarring [20]. A study by Fielding et al. identified IL-19 as another inflammatory marker involved in AV pathogenesis, expressed by epithelial cells and keratinocytes in response to proinflammatory stimuli [21,22]. Another study linked elevated serum IL-19 levels to AV severity, suggesting its potential role in disease progression [23]. Notably, IL-19 amplifies inflammation through a positive feedback loop, sustaining cytokine production [24], which may contribute to tissue damage and the progression of AV [23].

### 1.6. CRP and Salivary Biomarkers

C-reactive protein (CRP) is an acute-phase protein widely recognized as a marker of systemic inflammation [25]. However, despite evidence supporting its role, findings remain limited and inconsistent [26]. In acne pathogenesis, IL-1, IL-6, and tumor necrosis factor-alpha (TNF-α)—major inflammatory mediators—stimulate CRP production in the liver. Therefore, if the inflammatory response is sufficiently strong, CRP levels may also be elevated in AV [25]. A 2022 study reported significantly higher serum and salivary levels of both CRP and IL-1β in AV patients compared to the control group [27]. Similarly, a study by El-Taweel et al. found elevated serum CRP and hepcidin levels in AV patients, with CRP levels correlating with disease severity [28]. However, two studies conducted in 2014 and 2015 found no significant increase in CRP levels in acne vulgaris patients [29,30]. Numerous studies [23] have highlighted the potential value of saliva in monitoring overall health, diagnosing various oral and systemic disorders [31], and guiding dose adjustments by tracking therapeutic drug levels [32,33]. In laboratory testing, blood and urine are the most commonly used biological samples for research. However, saliva offers certain advantages over them [34]. Saliva collection is easy, non-invasive, and painless [35]. Additionally, saliva offers a practical alternative when blood sampling is difficult, such as in conditions like pemphigus vulgaris, where extensive skin erosion limits invasive procedures [36].

### 1.7. Aim of the Study

Building on existing evidence, this study examines the relationship between serum and salivary levels of CRP, IL-17, and IL-19 in patients with acne vulgaris (AV), with a particular focus on their association with disease severity and scarring forms. Moreover, it evaluates the potential of saliva as a non-invasive biomarker for monitoring cytokines and inflammatory activity in AV. Aim of this paper is to assess the potential of selected biomarkers in two different sampling methods to introduce a novelty for possible bioindicators in AV.

## 2. Results

When participants were grouped and compared based on acne status, serum IL-17 levels were found to be significantly higher in the control group compared to both the moderate and severe acne groups, regardless of gender (Table 1).

A similar comparison in terms of saliva showed no significant difference between the groups (Table 2).

Statistical comparisons were performed using one-way ANOVA followed by Tukey’s post hoc test.

When the three groups were reclassified into two categories—control and acne—a significant difference in serum IL-17 levels was observed, with the control group exhibiting higher IL-17 concentrations compared to the combined acne group (Table 3).

When these three groups were evaluated only as control and acne groups, no significant difference was observed in salivary values between the groups (Table 4).

When the participants were categorized based on acne severity (control, moderate, and severe) and gender (female and male), the data were statistically analyzed across six subgroups. The study groups were defined as follows: G1—Female Control (n = 18), G2—Female Moderate Acne (n = 35), G3—Female Severe Acne (n = 20), G4—Male Control (n = 10), G5—Male Moderate Acne (n = 8), and G6—Male Severe Acne (n = 8).In females, a significant difference in serum IL-17 was observed between the control and moderate groups, as well as between the control and severe groups (G1 vs. G2-G1 vs. G3). In males, a significant difference in serum IL-17 was observed between the control and moderate groups (G4 vs. G5). Serum IL-17 values were found to be higher in the control groups. No difference was observed in other parameters (Table 5).

When the same parameters were analyzed in saliva samples, no statistically significant differences were detected across the groups (Table 6).

When all participants (control and acne groups combined) were analyzed together, a statistically significant positive correlation was identified between salivary IL-17 and IL-19 levels (r = 0.672, *p* < 0.005), as well as between serum IL-17 and IL-19 levels (r = 0.538, *p* < 0.005).

No significant correlations were found between serum and saliva concentrations of CRP, IL-17, or IL-19, indicating that biomarker levels in saliva did not mirror those in serum. In addition, no correlations were observed between CRP and any of the interleukin parameters within or across sample types, further supporting that CRP did not exhibit any intra- or inter-compartment associations.

## 3. Discussion

AV is a disorder of the pilosebaceous unit with a complex and multifactorial etiology. In recent years, inflammation has been increasingly recognized as a key contributor to its pathogenesis [37]. This study examined serum and salivary levels of CRP, IL-17, and IL-19 in patients with moderate to severe AV to assess the inflammatory response. The comparative analysis of these biomarkers offers insights into the potential systemic involvement of inflammation in AV.

To evaluate effects of AV on inflammatory parameters, CRP was one of the assessed parameters. From a gender perspective in healthy individuals, previous studies have reported that CRP levels are generally higher in females than in males [38,39]. This difference has been attributed to the higher body fat percentage in females, which is associated with elevated systemic CRP concentrations indicative of chronic low-grade inflammation [39,40].

However, a recently published study challenges this traditional view, suggesting that the reference range for CRP in females should be assessed at a lower threshold compared to males [41]. In our study no significant difference in serum CRP levels was observed between the female and male control groups. El-Taweel et al. reported a correlation between CRP levels and acne vulgaris severity [28], whereas other studies have found no significant differences in CRP levels [29,30,42]. Consistent with several previous studies, our findings did not reveal a significant difference in CRP levels among control group and patients with moderate to severe acne vulgaris, regardless of gender. Salivary CRP levels showed no significant difference between males and females. Discrepancies in the literature may stem from differences in study design, patient demographics, disease severity, and the timing of measurements—particularly whether assessments were performed during an active inflammatory flare, when lesion inflammation is temporarily heightened.

Additionally, the lack of a severity-related increase in serum CRP—an established marker of systemic inflammation—indicates that the inflammatory activity in acne vulgaris remains largely local rather than systemic.

In addition to CRP, IL 19 was also evaluated. A study conducted by Mochtar et al. in 2018 reported that serum IL-19 levels increased with disease severity in patients with acne vulgaris [23]. Similarly, two studies found that serum IL-19 levels were significantly higher in acne vulgaris patients compared to the control group, with this increase being proportional to disease severity [43,44]. One of these studies reported elevated IL-19 levels in patients who had received recent systemic or topical acne treatments. Unlike our cohort—whose participants had not used any topical or systemic treatment for at least one month prior to enrollment and were screened according to detailed exclusion criteria—this treatment exposure may have influenced cytokine expression and contributed to the differences observed in IL-19 levels between the studies [44].

Furthermore, a recent study evaluating serum IL-19 concentrations in acne vulgaris reported elevated IL-19 levels compared to healthy controls, reinforcing the literature linking IL-19 to acne-related inflammation; however, no significant correlation was observed between IL-19 levels and acne severity [45].

However, our study found no significant differences in serum and salivary IL-19 levels between patients with moderate and severe AV and the control group. Similarly, no significant differences were observed between males and females across study groups. Notably, none of the participants had received systemic or topical acne treatment in the past two months. This discrepancy may be related to differences in treatment history, study populations, or the timing of sample collection. In our cohort, neither IL-19 nor CRP demonstrated a severity-related systemic increase, supporting the interpretation that the inflammatory activity in acne vulgaris is predominantly localized rather than strongly systemic.

IL-17 was also evaluated in this current study. In 2014, Kelhälä et al. reported a substantial elevation in IL-17 levels within acne lesions compared to skin biopsies from healthy individuals [16]. A case–control study found significantly higher IL-17 expression in both acne lesions and non-lesional epidermis of acne patients compared to healthy subjects [46]. Recent immunological analyses have further emphasized the diverse cellular contributors to early acne inflammation—including keratinocytes, macrophages, neutrophils, and Th17-related pathways—underscoring the multifaceted cytokine environment involved in acne pathogenesis [47]. In the study by Ebrahim et al., serum IL-17 levels were reported to be significantly higher in acne vulgaris patients compared to the control group, with this increase being associated with disease severity [20]. Similarly, Murlistyarini et al. reported a positive correlation between IL-17 levels and the severity of AV [48]. More recent findings further support this pattern; Al-Rubaye et al. demonstrated that both IL-17A and IL-17F levels were significantly elevated in acne patients, particularly in more severe cases, indicating broader activation of the Th17 pathway in advanced disease [49].

In contrast, Topan et al. reported no significant difference in serum IL-17 levels between acne patients and healthy controls and found no association between IL-17 levels and acne severity [50]. In our study, IL-17 levels showed significant differences between the control and acne groups; however, serum IL-17 levels were unexpectedly lower in acne patients compared to the control group. While research on IL-17 in acne vulgaris has primarily focused on tissue measurements, studies examining serum IL-17 levels have produced conflicting results. Most previous studies have reported a positive correlation between AV severity and serum IL-17 levels, whereas our findings suggest a different pattern, possibly influenced by regulatory immune mechanisms or disease stage. Although IL-17 is known to contribute to acne-related inflammation, [51] it also plays a complex role in immune regulation. It helps maintain balance between the host and commensal microbiota, supporting barrier integrity and microbial homeostasis. However, its effects can be both protective and detrimental [52]. Imbalanced IL-17 activity can trigger chronic inflammation and autoimmunity, highlighting its crucial role in maintaining immune homeostasis [53]. In healthy skin, IL-17 is produced by multiple immune cell subsets—including Th17 cells, Tc17 cells, γδ T cells, and innate immune cell populations—with Tc17 cells being one of the microbiota-responsive sources that help regulate microbial communities and provide antifungal protection [54,55].

Therefore, higher IL-17 levels in healthy individuals may reflect its essential function in preserving skin homeostasis. Recent studies highlight IL-17’s protective role in promoting wound healing and mitigating physiological stress [56]. On the other hand therapeutic targeting of IL-17 in AV has shown mixed results. A clinical trial on anti-IL-17A therapy for moderate to severe acne found no significant reduction in inflammatory lesions compared to a placebo [51]. Altogether chronic inflammation in acne may activate feedback mechanisms that reduce systemic IL-17 to prevent excessive immune response. Increased regulatory Tcell (Treg) activity could also suppress Th17-driven IL-17 production, lowering serum levels despite skin inflammation. Saliva offers significant advantages as a non-invasive biomarker tool. Its easy and painless collection makes it a practical option, particularly for pediatric patients or individuals for whom invasive procedures are not preferred. Studies suggest that saliva can be used to monitor inflammatory markers and that cytokines such as IL-17 and IL-19 in saliva may reflect systemic inflammation [27]. However, in our study, no significant differences were found in CRP, IL-17, and IL-19 levels between groups in saliva samples. The significant correlations observed within both saliva and serum suggest a potential interaction between IL-17 and IL-19 in immune regulation. As both cytokines play key roles in inflammatory processes—particularly in Th17-mediated responses—the consistency of these correlations indicates that salivary cytokine levels may reflect systemic cytokine activity and serve as potential non-invasive biomarkers [57,58].

Taken together, CRP, IL-19, and IL-17 display a consistent pattern in our cohort: none showed severity-related systemic elevation despite their known local roles in acne pathophysiology. This collective profile supports the concept that acne vulgaris involves primarily localized rather than systemic inflammation and that mechanistic interactions between IL-17 and IL-19 may occur at the skin level without substantial systemic reflection.

This study has some limitations such as sample size, single time-point measurement, and possible confounding factors (diet, circadian variation, menstrual cycle) which can be investigated in future studies.

## 4. Materials and Methods

### 4.1. Experimental Design and Participants

This study included a total of 99 participants. 71 volunteers diagnosed with Acne Vulgaris of varying severity (ages 15–30) and a control group. The control group (n = 28) consisted of healthy individuals without acne vulgaris, confirmed through dermatological examination, and served as the negative control for all comparisons in the study. The sample size was determined based on feasibility and recruitment availability within the study period and was confirmed as adequate through post hoc statistical power evaluation to ensure sufficient reliability of the main comparisons.

Ethical approval for this study was obtained from the Ethics Committee for Human Research at Hacettepe University Faculty of Medicine (Ankara, Türkiye) (Approval No: GO 22/876; Approval Date: 18 October 2022), and all procedures were conducted in accordance with the Declaration of Helsinki.

The research details were explained to all participants, and informed consent was obtained. For those under 18 years of age, both child assent and legal guardian consent forms were provided. Additionally, participants filled out personal declaration forms containing information about their general health status and medication use. Exclusion criteria included: history of hormonal therapy or isotretinoin treatment; pregnancy or lactation; presence of systemic or inflammatory diseases; poor oral hygiene, periodontal disease, or oral injuries; and a history of smoking, alcohol use, or substance addiction.

Acne severity was assessed using the Global Evaluation Acne (GEA) Scale developed by Dréno et al. which categorizes severity from 0 (no lesions) to 5 (very severe acne) based on lesion type (open/closed comedones, papules, pustules, nodules) and facial involvement [59].

In our study, participants were classified into three groups—Control (n = 28), Moderate Acne (n = 43), and Severe Acne (n = 28)—based on the GEA scale under dermatological evaluation. Participants in the control group were additionally examined by a dermatologist to confirm the complete absence of acne lesions.

### 4.2. Biochemical Analyses

For biochemical analysis, 5 mL of venous blood and 5–10 mL of saliva samples were collected from each participant. For individuals with insufficient saliva production, samples were obtained by chewing paraffin film. A 5 mL venous blood sample was collected from each participant by standard venipuncture, and the blood was placed into tubes without anticoagulant for serum separation.

All biological samples were initially stored at +4 °C for 30 min and subsequently centrifuged at +18 °C to obtain serum and isolate salivary particulates. Blood samples were centrifuged at 5000 rpm for 15 min, while saliva samples were centrifuged at 4000 rpm for 7 min. Post-centrifugation, serum from blood samples and supernatant from saliva samples were transferred into separate Eppendorf tubes and stored at −20 °C.

For the quantification of interleukin-17 (IL-17) and interleukin-19 (IL-19), frozen samples were gradually thawed on ice and subsequently brought to room temperature before analysis via enzyme-linked immunosorbent assay (ELISA). Serum CRP levels were measured through outsourced biochemical analysis performed at Baran Medical Laboratory (Ankara, Türkiye).

Total protein concentration was determined using the Bicinchoninic Acid (BCA) assay, following the original method described by Smith et al. (1985) [60]. Protein determination was performed using a 96-well BCA reagent kit (Cat. No. E-BC-K318-M; Elabscience Biotechnology Co., Ltd., Wuhan, China), and all steps were carried out strictly according to the manufacturer’s protocol. A 200 µL sample volume was added to each well, and absorbance was measured at 562 nm using a microplate reader. Protein concentrations were calculated from the kit-provided standard curve, and the mean protein concentration was recorded. After obtaining the results from the BCA assay, a 3-fold dilution with distilled water was deemed appropriate before analysis to reduce sample concentration and enhance measurement sensitivity. Human IL-17 and IL-19 levels in saliva and serum were quantified using BT Lab ELISA kits (IL-17: Cat. No. EO142Hu; IL-19: Cat. No. E3929Hu; Bioassay Technology Laboratory, Shanghai, China), in accordance with the manufacturer’s instructions. After obtaining the BCA assay results, a uniform 1:3 dilution with distilled water was applied to all samples to ensure that cytokine concentrations fell within the optimal detection range of the ELISA kits. The prepared samples and standards were loaded onto 96-well microplates, and all subsequent steps were carried out as per protocol. Optical density (OD) was measured at 450 nm using a microplate reader. Cytokine concentrations were calculated using the respective standard curves.

The CRP concentrations in saliva samples were quantified using the Otto Scientific CRP ELISA kit (Cat. No. OttoBC138; Otto Scientific, Ankara, Türkiye), based on an immunoturbidimetric colorimetric method. Measurements were performed using the MINDRAY-BS400 autoanalyzer (Mindray Bio-Medical Electronics Co., Ltd., Shenzhen, China), and CRP concentrations were calculated from a standard curve. Serum CRP levels were measured at Baran Medical Laboratory (Ankara, Türkiye).

### 4.3. Statistical Analysis

All collected data were securely stored on the principal investigators’ computers and analyzed using SPSS version 16.0 (IBM Corp., Armonk, NY, USA). The Shapiro–Wilk test was used to assess normality, mean, standard deviation were calculated for numerical variables, and group comparisons were performed using one-way ANOVA followed by Tukey’s or Duncan’s post hoc tests. A *p*-value < 0.05 was considered statistically significant.

## 5. Conclusions

Although the sample size in our study was adequate, natural biological variability among participants—including factors not assessed in this study, such as BMI or detailed dietary habits—may have contributed to differences in cytokine levels. Additionally, evaluating cytokine concentrations at a single time point and only at the systemic level may not fully capture the dynamic inflammatory processes underlying acne pathophysiology.

The unexpected finding of higher serum IL-17 levels in the control group challenges conventional perspectives and underscores the complexity of immune regulation in acne. This observation suggests that IL-17 may have context-dependent roles that warrant further investigation. Future studies should aim to clarify the mechanistic significance of IL-17 in acne development, as well as its potential diagnostic or therapeutic usefulness. Comprehensive cytokine profiling, longitudinal monitoring of systemic IL-17 levels, and concurrent evaluation of both local (skin) and systemic inflammatory markers are needed to provide a more integrated understanding of acne-related immune pathways.

In addition, incorporating larger, demographically diverse cohorts, assessing lifestyle-related variables, and integrating tissue-level analyses—such as skin biopsies or non-invasive sampling techniques—may help refine the interpretation of cytokine dynamics. These efforts could contribute to the development of more precise biomarkers and support the advancement of personalized therapeutic strategies in acne management.

Findings of this study produce both information about selected parameters to this common disease as well as some questions to be addressed. Effects of time course during sampling providing multiple time-point measurements as well as possible confounding factors (diet, circadian variation, menstrual cycle) can be investigated in future studies with a greater sample size.

## Figures and Tables

**Table 1:** Serum CRP (mg/L), IL-17 (ng/L), and IL-19 (ng/L) levels measured using immunoturbidimetric assay (CRP) and ELISA (IL-17, IL-19) across study groups classified by acne severity using the Global Evaluation Acne (GEA) Scale.

Serum	Control	Moderate	Severe
CRP [mg/L]	2.25 ± 2.74	2.02 ± 2.30	1.69 ± 3.44
IL 17 [ng/L]	286.09 ± 188.37 *	199.41 ± 139.76	181.95 ± 96.25
IL 19 [ng/L]	110.46 ± 50.56	95.05 ± 50.40	94.18 ± 63.05

Results are presented as mean ± standard deviation. Control group (n = 28), Moderate Acne (n = 43), Severe Acne (n = 28). n represents the number of participants in each group. Statistical comparisons were performed using one-way ANOVA followed by Tukey’s post hoc test. * indicates *p* < 0.05 for comparisons between the Control group and both Moderate and Severe acne groups.

**Table 2:** Salivary CRP (mg/L), IL-17 (ng/L), and IL-19 (ng/L) levels measured using immunoturbidimetric assay (CRP) and ELISA (IL-17, IL-19) across study groups classified by acne severity using the Global Evaluation Acne (GEA) Scale.

Saliva	Control	Moderate	Severe
CRP [mg/L]	0.39 ± 0.09	0.34 ± 0.11	0.36 ± 0.10
IL 17 [ng/L]	144.42 ± 39.72	134.53 ± 48.21	139.35 ± 59.03
IL 19 [ng/L]	93.33 ± 26.35	90.37 ± 21.19	86.13 ± 28.63

Results are presented as mean ± standard deviation. Control group (n = 28), Moderate Acne (n = 43), Severe Acne (n = 28). n represents the number of participants in each group.

**Table 3:** Serum CRP (mg/L), IL-17 (ng/L), and IL-19 (ng/L) levels in the Control group and the combined Acne group (Moderate + Severe), without distinguishing acne severity.

	Control	Acne
CRP (mg/L)	2.25 ± 2.74	1.89 ± 2.79
IL 17 (ng/L)	286.09 ± 188.37 *	192.53 ± 123.96
IL 19 (ng/L)	110.46 ± 50.56	94.71 ± 55.29

Results are given as mean ± standard deviation. Control (n = 28), combined Moderate + Severe Acne (n = 71). Statistical comparisons were performed using one-way ANOVA followed by Tukey’s post hoc test. * indicates *p* < 0.05, representing a significant difference between the Control and Acne groups.

**Table 4:** Salivary CRP (mg/L), IL-17 (ng/L), and IL-19 (ng/L) levels in the Control group and the combined Acne group (Moderate + Severe), without distinguishing acne severity.

	Control	Acne
CRP [mg/L]	0.39 ± 0.09	0.35 ± 0.11
IL 17 [ng/L]	144.42 ± 39.72	136.43 ± 52.38
IL 19 [ng/L]	93.33 ± 26.35	88.70 ± 24.29

Results are given as mean ± standard deviation. Control (n = 28), combined Moderate + Severe Acne (n = 71). Statistical comparisons were performed using one-way ANOVA followed by Tukey’s post hoc test.

**Table 5:** Serum CRP (mg/L), IL-17 (ng/L), and IL-19 (ng/L) levels in female and male participants across acne severity groups classified according to the Global Evaluation Acne (GEA) Scale.

Serum			
Female	G1 Control	G2 Moderate	G3 Severe
CRP [mg/L]	2.53 ± 3.04	1.52 ± 1.54	1.01 ± 0.67
IL 17 [ng/L]	315.20 ± 224.45 *	211.69 ± 151.51	92.36 ± 89.90
IL 19 [ng/L]	115.27 ± 53.53	95.76 ± 54.45	87.52 ± 29.32
Male	G4 Control	G5 Moderate	G6 Severe
CRP [mg/L]	1.75 ± 2.17	4.21 ± 3.66	3.41 ± 6.31
IL 17 [ ng/L]	236.61 ± 92.91 ^+^	145.69 ± 40.67	173.35 ± 116.96
IL 19 [ ng/L]	101.81 ± 46.12	91.93 ± 28.81	110.84 ± 112.05

Results are given as mean ± standard deviation. The study subgroups were composed as follows: G1—Female Control (n = 18), G2—Female Moderate Acne (n = 35), G3—Female Severe Acne (n = 20); G4—Male Control (n = 16), G5—Male Moderate Acne (n = 8), G6—Male Severe Acne (n = 8). n represents the number of participants in each subgroup. Statistical comparisons were performed using one-way ANOVA followed by Tukey’s post hoc test, with *p* < 0.05 considered statistically significant. * indicates *p* < 0.05, representing a significant difference. Serum IL-17 values were higher in the control group compared to the moderate and severe acne groups. ^+^ indicates *p* < 0.05 indicates a significant difference. Serum IL-17 values were found to be higher in the control group (Male).

**Table 6:** Comparison of salivary results of individuals divided into 6 groups based on gender and acne status.

Saliva			
Female	G1 Control	G2 Moderate	G3 Severe
CRP [mg/L]	0.39 ± 0.11	0.35 ± 0.12	0.37 ± 0.08
IL 17 [ng/L]	157.29 ± 32.77	138.10 ± 47.84	129.04 ± 63.24
IL 19 [ng/L]	94.79 ± 26.36	92.88 ± 20.63	81.59 ± 26.33
Male	G4 Control	G5 Moderate	G6 Severe
CRP [mg/L]	0.38 ± 0.06	0.30 ± 0.09	0.34 ± 0.14
IL 17 [ng/L]	121.25 ± 42.15	118.93 ± 49,86	165.15 ± 39.01
IL 19 [ng/L]	90.42 ± 27.69	79.41 ± 21.40	97.49 ± 32.79

Results are given as mean ± standard deviation. The study subgroups were composed as follows: G1—Female Control (n = 18), G2—Female Moderate Acne (n = 35), G3—Female Severe Acne (n = 20); G4—Male Control (n = 16), G5—Male Moderate Acne (n = 8), G6—Male Severe Acne (n = 8). n represents the number of participants in each subgroup. Statistical comparisons were performed using one-way ANOVA followed by Tukey’s post hoc test, with *p* < 0.05 considered statistically significant.

## Data Availability

The original contributions presented in this study are included in the article. Further inquiries can be directed to the corresponding author.

## Abbreviation

The following abbreviations are used in this manuscript:

AV	Acne vulgaris
CRP	C-reactive protein
IL-17	Interleukin-17
IL-19	Interleukin-19
TLRs	Toll-like receptors
Th17	T helper 17 cells
IFN-γ	Interferon-gamma
TNF-α	Tumor necrosis factor-alpha
ELISA	Enzyme-linked immunosorbent assay
OD	Optical density
BCA	Bicinchoninic acid assay
GEA	Global Evaluation Acne (Scale)
Treg	Regulatory T cell
SPSS	Statistical Package for the Social Sciences
